# Perceptions, attitudes, and behaviors of asthma patients towards the use of short-acting β_2_-agonists: A systematic review

**DOI:** 10.1371/journal.pone.0283876

**Published:** 2023-04-20

**Authors:** Zhe Chi Loh, Rabia Hussain, Shamala Balan, Bandana Saini, Jaya Muneswarao, Siew Chin Ong, Zaheer-Ud-Din Babar

**Affiliations:** 1 School of Pharmaceutical Sciences, Universiti Sains Malaysia, Gelugor, Pulau Pinang, Malaysia; 2 Pharmacy Department, Hospital Tengku Ampuan Rahimah, Klang, Selangor, Malaysia; 3 Faculty of Medicine and Health, The University of Sydney, Sydney, Australia; 4 Pharmacy Department, Hospital Pulau Pinang, George Town, Pulau Pinang, Malaysia; 5 Department of Pharmacy, University of Huddersfield, Huddersfield, United Kingdom; University of Sheffield, UNITED KINGDOM

## Abstract

**Background:**

Short-acting β_2_-agonists (SABA), the most potent and rapid-acting relievers are commonly used to provide quick relief of asthma symptoms. However, there is an increasing concern regarding the misuse of SABA medicines.

**Objective:**

This qualitative systematic review aims to determine, evaluate, and summarize the perceptions, attitudes, and behaviors towards the use of SABA from the patients’ perspectives.

**Methods:**

The databases searched included PubMed, Scopus, PsycINFO, CINAHL, and Cochrane database. Original research articles reporting the perceptions, attitudes, or behaviors of asthma patients towards the use of SABA, which was available as full text, published in the English language between the year 2000 and February 2023 were included in the review. Commentaries, letters to editor, review articles, and conference proceedings were excluded.

**Results:**

A total of five articles were included. Six overarching themes were obtained: (1) perceptions on health status; (2) perceptions and attitudes towards the impact of asthma; (3) perceptions towards asthma control; (4) perceptions towards asthma knowledge; (5) risk perceptions; (6) perceptions, attitudes, and behaviors towards the use of SABA.

**Conclusion:**

Despite the fact that SABA could rapidly alleviate asthma symptoms, SABA over-users were less likely to describe their health status and asthma control as ‘excellent’. Most SABA over-users did not know that frequent SABA usage would worsen their asthma control, and they exhibited psychological linkage towards the use of SABA. Collaborative efforts between policymakers, healthcare professionals and patients are warranted to reconstruct SABA prescribing practice and usage.

## Introduction

Asthma is clinically defined as ‘the history of respiratory symptoms such as wheeze, shortness of breath, chest tightness, and cough that vary over time and in intensity, together with variable airflow limitation [1]. Globally, data reported by the World Health Organization highlighted 455,000 deaths attributable to asthma with 339 million people living with the condition [2, 3]. Only 4.3% of these asthma cases were doctor-diagnosed [4]. In the year 2019, 84.5% of asthma patients with current asthma and 78.7% of those who have ever been diagnosed with asthma reside in low- and middle-income countries [5]. In France, about 6.4% of patients with current asthma were reported in the year 2018 [6]. Additionally, 5.9%, 9.5%, 4.6%, and 5.5%, were reported as the prevalence rates of asthma in Germany, the United Kingdom, Italy, and Spain respectively [7]. The prevalence of asthma cases was about 6.5% in the United States, while China had recorded about 9.8% of adult asthma cases [8, 9]. In Malaysia, acute asthma attacks, emergency department visits, and asthma hospitalizations were recorded to be 67.8%, 20.0%, and 10.0%, respectively [10]. Pakistan, on the other hand, had an asthma prevalence rate of 4.3% [11].

The underlying pathophysiology of asthma is an inflammatory process that results in airway hyper-responsiveness, bronchoconstriction, and other symptoms. Most clinical management guidelines aim to improve disease control. Asthma control is defined by two domains: symptom control and future risk of adverse outcomes (e.g., exacerbations or loss of lung function) [1]. Asthma medications are mainly categorized into ‘relievers’ and ‘preventers’ based on their mechanism of action. Relievers include medications that relieve bronchoconstriction whilst preventers have an anti-inflammatory mode of action. Salbutamol is the most common reliever used globally [1]. It is quite specific to β_2_ adrenergic receptors, and when used in an inhalation form, can bring about bronchodilation by relaxing the bronchial smooth muscles, rapidly, within three to five minutes, and its effects last from four to six hours [12]. Before the year 2019, SABAs were recommended as monotherapy on an as-needed basis (*pro re nata or prn*) in most earlier guidelines for patients with very mild asthma, with infrequent symptoms [13]. However, the Global Initiative for Asthma (GINA) 2019 guidelines, in a paradigm shift, no longer recommend the use of SABA monotherapy for mild asthma patients [1]. This shift is attributable to increased concerns around adverse asthma outcomes when SABAs are overused, especially when used without anti-inflammatory preventer medications in those with asthma [1, 13]. Patients with well-controlled asthma should need to use doses from a SABA reliever inhaler less than twice per week [1]. Needing more doses implies that the condition is not well controlled and should signal a treatment review.

As mentioned above, SABA overuse has been associated with an increased risk of poor asthma control, hospitalization, and asthma-related death [13–16]. Evidence suggests that using ≥ three or more canisters of a SABA inhaler in a year (average 1.6 puffs per day) is associated with an increased risk of flare-ups [17]. Using ≥12 or more canisters of a SABA inhaler in a year (average 6.6 puffs per day) is associated with an increased risk of asthma death [18].

SABA overuse (collecting ≥ three SABA inhalers a year) is quite prevalent globally [19]. The SABINA II (SABA use IN asthma) main study, which collected and analyzed prescription records for patients diagnosed with asthma across many European countries indicated SABA overuse in countries ranging from 9% (Italy), 16% (Germany), 29% (Spain), 30% (Sweden), and 38% (United Kingdom (UK)) [20]. In the UK, SABA overuse was nearly twice as prevalent in those with moderate-severe versus mild asthma [15]. The SABINA Canada study similarly highlighted a high prevalence of SABA overuse across the provinces of Nova Scotia and Alberta (28% and 39.4% of those with asthma) [21]. This high prevalence is also seen in Asian countries, for example, in Taiwan, 15.9% of asthma patients were reported to be SABA over-users using a pay-for-performance database (Taiwan P4P Asthma Program) [22]. In Malaysia, as part of the SABINA III study, data highlight that, about 47.4% of asthma patients were prescribed three or more SABA canisters per year and 17.7% of asthma patients were prescribed 10 or more SABA canisters per year [14].

In people with asthma, SABA overuse, which includes SABA monotherapy and excessive doses, may occur for myriad reasons, for example, the perception of immediate relief compared to using preventers, use for a long time where people may deem it safe, and availability over the counter in some countries.

Indeed, the perceptions, attitudes, and behaviors of patients toward a specific healthcare issue are paramount in understanding and identifying disease management strategies [23]. To minimize SABA overuse, national-level strategies and campaigns may be required. Clearly, such campaigns or messages have not been effective as SABA overuse is an ongoing risk. Risk communication experts suggest that risk perceptions and attitudes (e.g., risk tolerance) drive behaviors. Therefore, risk communication messages should be informed by a collected understanding of the risk perceptions and attitudes that drive SABA use related risk behaviors in people with asthma.

To the best of our knowledge, there is no global synthesis of data that can inform SABA related risk communication messages. This systematic review, therefore, is aimed at determining, evaluating, and summarizing the findings of perceptions, attitudes, and behaviors towards the use of SABA from the asthma patients’ perspectives. This data, along with guiding principles based on behavioral and cognitive science could facilitate the development of effective risk communication strategies for healthcare providers managing patients’ overusing SABAs and for shifting perceptions about the risks related to SABA overuse in asthma patients. Additionally, the perceptions, attitudes, and behavior of the patients toward the use of SABA may offer crucial and comprehensive details concerning the standard of asthma care management [24].

## Materials and methods

### Eligibility criteria

The definitions used in this systematic review were:

#### Perceptions

The understanding or beliefs about a phenomenon based on previous experience, culture, and information processing [25, 26]. Additionally, knowledge, which is a form of stored meanings of previous visual experiences, is intimately tied to individuals’ perceptions [27].

#### Attitudes

A tendency to respond positively or negatively towards a certain idea, object, person, or situation [28].

#### Behaviors

The activities taken by a person who believes himself to be healthy or sick so that he or she can detect, prevent, or recover from the disease [29]. Social processes may have an impact on how particular behaviors develop through time, which in turn shapes the formation of practices [30].

The inclusion criteria were English language original research articles reporting the perceptions, attitudes, or behaviors of asthma patients towards the use of SABA. These full text articles were published between the year 2000 to February 2023.This systematic review was not limited to any specific study designs. The commentaries, letters to editor, review articles, and conference proceedings were not included in this systematic review.

### Information sources and search strategy

This systematic review was conducted in accordance with The Preferred Reporting Items for Systematic Reviews and Meta-Analyses (PRISMA) guidelines [31, 32]. The literature was searched by utilizing the “Boolean operators” such as “AND” or “OR” to combine the following keywords: *perception*, *belief*, *perspective*, *knowledge*, *understanding*, *attitude*, *respond*, *behavior*, *practice*, *short-acting β*_*2*_*-agonist*, *SABA*, *terbutaline*, *reliever*, *salbutamol*, *albuterol*. The search strategies applied in different databases were displayed in S1 Table.

The combinations were used on the PubMed platform, and this step was repeated at other databases such as Scopus, PsycINFO, CINAHL, and Cochrane database, from the year 2000 –February 2023. These subject-specific and multidisciplinary databases were selected due to their easy accessibility and availability of publications on life science and biomedical subjects [33]. The reference lists of relevant studies were also reviewed to further identify related articles and prevent missing information.

### Selection process

One author (ZCL) carried out the selection process for eligible studies to be included in this systematic review and was counter-checked by the other two authors (SB and RH). First, the titles and abstracts of the studies identified from different databases were screened [34–36]. Full text articles were assessed when the titles and abstracts were insufficient to provide the relevant information. The full text articles which did not meet the inclusion criteria were excluded. The studies were selected based on relevance and acceptability [37, 38]. Furthermore, the explicit method which emphasized following the inclusion criteria was strictly applied to ensure the quality of the selection process [39]. No automation tools were utilized for the articles’ selection process. The author (ZCL) consulted an experienced pharmacist (SB) and a senior lecturer (RH) from a university to clarify doubts during the articles’ selection process.

### Data collection

The data were extracted by two independent authors (ZCL and RH) into a data extraction sheet using Microsoft Excel. The information extracted from the selected studies included country, study setting and the number of center(s), study design, study tool, characteristics of participants and sample size, key findings, limitations, and recommendations.

### Quality assessment

Trustworthiness, relevance, and results of the included studies were assessed by using the Joanna Briggs Institute (JBI) critical appraisal checklists which were obtained from the official website [40, 41]. Two authors (ZCL and RH) assessed each study independently. The JBI checklist for qualitative research and JBI checklist for analytical cross-sectional analysis were utilized for the quality assessments, as shown in the supplementary material S2 and S3 Tables, respectively. The quality assessment checklists applied a scoring system, whereby one point was allocated for the study which fulfilled the stated criteria of the checklist. Otherwise, zero point was allocated when the study did not satisfy the selected criteria. The quality percentage was calculated using the formula below:

$$Quality\;percentage=\frac{Sum\;of\;allocated\;point\;from\;each\;stated\;criteria}{Total\;number\;of\;stated\;criteria}\times 100\%$$

Quality percentage of 0–33% was ranked as low quality, 34–66% was ranked as medium quality, while 67% or more will be ranked as high quality [42]. Any disagreement between the two authors (ZCL and RH) in conducting the quality assessment was resolved by discussion and consensus.

### Data synthesis and analysis

All the included studies in this research were evaluated through thematic analysis to synthesize the key findings from the data [43–45]. Whereby, thematic analysis involves reading texts and identifying key findings that capture the overall meaning of the text [46, 47]. The key findings were described in a way, that the common points across all eligible studies were summarized in the form of overarching themes [47–49]. Overarching themes that emerged from this study allowed a comprehensive and nuanced understanding of the key findings across all eligible studies. The statistical data were included to indicate the magnitude of the key findings, it was not used in the data synthesis process. As a result, handling of any missing summary statistics was not needed in this systematic review.

### Outcome

The outcomes of the current systematic review were the perceptions, attitudes, and behaviors towards the use of SABA.

## Results

**Fig 1:**
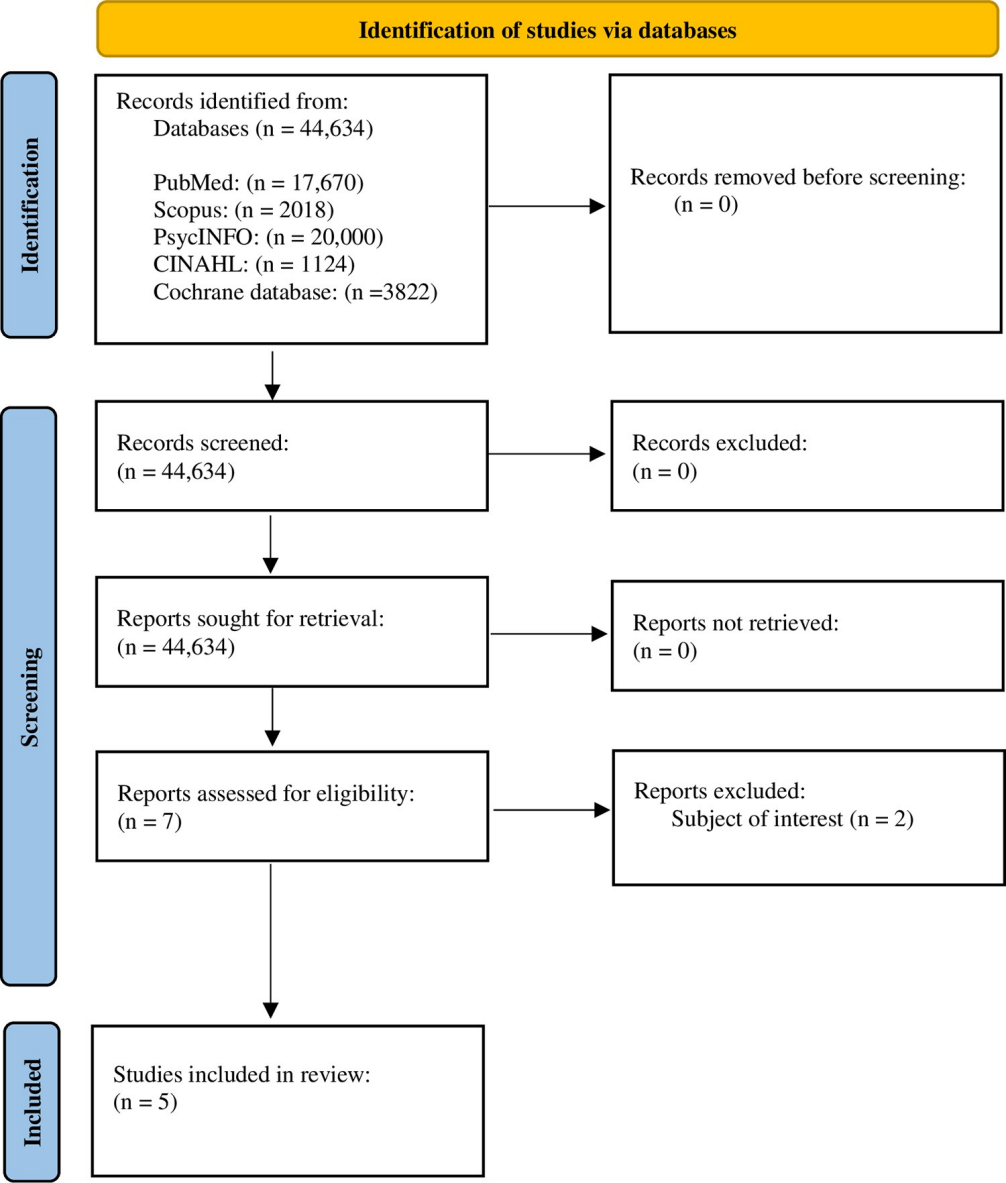
PRISMA flow diagram for the screening and selection of studies.

After conducting an electronic search, a total of 44,634 results were obtained, 17,670 searches were from PubMed, 2018 searches from Scopus, 20,000 searches from PsycINFO, 1124 searches from CINAHL, and 3822 searches from Cochrane database. Out of these, all titles and abstracts were screened, and if any study was found relevant, it was subjected to full-length assessments. However, after the screening of 44,634 studies, only seven studies underwent a full-length assessment based on the inclusion criteria. Out of the seven studies, only five studies met the eligible criteria, while two studies were excluded due to lacking in the subject of interest. The process of study selection was displayed in a flowchart diagram as shown in Fig 1. The flowchart diagram was obtained from the official website of PRISMA [32].

Two studies reported findings in Australia, one study reported data from the United States, one study was conducted in the United Kingdom and another study was carried out in Canada, France, Germany, Japan, the United Kingdom, and the United States [50–54] (Table 1). After quality assessments, all the studies were deemed to be of ’high quality’.

**Table 1:** Characteristics of studies included in this systematic review.

No.	Reference	Country/ Countries	Study setting and no. of center(s)	Study design	Study tool	Characteristics of participants	Sample size
1.	(Hong et al., 2006) [52]	The United States	Household components	Retrospective analysis (cross-sectional analysis)	Medical Expenditure Study survey	Asthma patients with five years and above who had used short-acting β_2_-agonists (SABA) from year 1996 to 2000 were included, whereas children less than five years old and chronic obstructive pulmonary disease patients were excluded.	2386
2.	(Azzi et al., 2019) [51]	Australia	18 different community pharmacies	Real-world cross-sectional observation study	Self-administered questionnaire	Individuals aged 16 years old and above, purchased SABA over the counter from community pharmacies, able to speak English, and no exclusion criteria were applied.	412
3.	(Cole et al., 2013) [53]	The United Kingdom	A large urban general practice. Computerized medical records were searched.	Qualitative study using a purposive extreme case sample	Interview	Asthma patients aged 20–32 years old, prescribed with short-acting bronchodilators in the last three months	21
4.	(Blakeston et al., 2021) [54]	Canada, France, Germany, Japan, the United Kingdom, and the United States	Patient panel, healthcare professional’s referral and finders	Multinational qualitative study	Interview	Asthma patients aged 18 years old and above, who had received short-acting β_2_-agonists (SABA) therapy either with or without maintenance therapy	80
5.	(Azzi et al., 2022) [50]	Australia	Community pharmacies	Real-world cross-sectional observation study	Questionnaire	Individuals aged 16 years old and above, purchased SABA over the counter from community pharmacies and able to speak English. No exclusion criteria were applied	412

Six overarching themes were obtained:

- Perceptions on health status
- Perceptions and attitudes towards the impact of asthma
- Perceptions towards asthma control
- Perceptions towards asthma knowledge
- Risk perceptions
- Perceptions, attitudes, and behaviors towards the use of short-acting β_2_-agonists

Different key findings as shown in Table 2, were defined as below:

- Health status: The patients’ perceptions or ideas regarding their overall health. Any type of scale could be used. The respondents could either rate their status as ‘excellent/very good, good, fair/poor’ or ‘excellent, very good, good, fair, or poor.
- Impact of asthma: Any limitations caused by asthma, which may include walking, social functioning, cognitive function, social and personal lives, frightening experience, the integrity of mental health, etc.
- Asthma control: The patients’ perceptions of asthma control (uncontrolled, well, or partly controlled) and the asthma control which is assessed by GINA criteria, asthma control test, or other validated tools.
- Asthma medication: The comments of asthma patients on asthma medication, particularly SABA. This may include when they use their medication, reasons they did not adhere to their medications, their feelings about the use of SABA relievers, etc.
- Asthma-related information: The comments of asthma patients regarding their own asthma knowledge.
- Risk perception: Patients’ subjective abilities to assess the risk associated with their usage of SABA and asthma based on how likely it is to occur and how serious the effects will be [55].

**Table 2:** Key findings, limitations, and recommendations.

No	Reference	Key findings	Limitation(s)	Recommendation(s)
1.	(Hong et al., 2006) [52]	*Health status*: Short-acting β2-agonists (SABA) inappropriate users were less likely to rate their health condition as ‘very good’ or ‘excellent’ compared with appropriate SABA users. *Impact of asthma*: SABA inappropriate users had an increased tendency to report functional limitations (daily living, walking, social and cognitive function) caused by asthma than appropriate users.	There is a possibility of patients who had written prescriptions for short-acting β_2_-agonists (SABA) and inhaled corticosteroids (ICS) but chose to fill only the SABA prescription.	Identifying the factors of misusing SABA.
2.	(Azzi et al., 2019) [51]	*Asthma control*: Short-acting β_2_-agonists (SABA) over-users have a higher tendency to develop uncontrolled asthma based on Global Initiative for Asthma (GINA)-defined criteria than SABA non-over-users. *Risk perceptions*: More SABA over-users experienced SABA-associated side effects than SABA non-over-users. *The use of SABA*: Short-acting β_2_-agonists (SABA) over-users had more SABA inhalers at any one time than SABA non-over-users. More SABA non-over-users used SABA when their symptoms present.	No doctor-confirmed asthma diagnosis. No response rate was captured. Seasonality that causes SABA overuse could not be identified.	Exploring how community pharmacists can better identify patients with uncontrolled asthma through the development of tools and strategies.
3.	(Cole et al., 2013) [53]	*Impact of asthma*: Asthma affected short-acting bronchodilators users’ daily lives, childhood, employment opportunities. *Asthma control*: High short-acting bronchodilators users had poor control of asthma symptoms. *The use of SABA*: Short-acting bronchodilators users felt embarrassed to use their inhalers. Besides, high short-acting bronchodilators users would prepare several inhalers to ensure they can have immediate access to them during asthma emergency attacks. Furthermore, the short-acting bronchodilators over-users were satisfied with the quick fix provided by the inhalers.	The findings cannot be generalized to a large population as the study was conducted in the urban general practice.	Evaluating the strategies to improve asthma control among bronchodilators over-users.
4.	(Blakeston et al., 2021) [54]	*Asthma knowledge*: Japanese respondents understand the reliever medication better than the respondents from Canada, France, German, the United Kingdom, and the United States. *The use of SABA*: Asthma patients were psychologically attached to SABA due to its efficacy and quick-relieve effects.	The results cannot be generalized to the larger population due to the small sample size.	Exploring more perceptions of asthma patients to the SABA usage and their treatment barriers.
5.	(Azzi et al., 2022) [50]	*Health status*: High SABA users were less likely to rate their overall health as ‘excellent/ very good’. *Impact of asthma*: High SABA users were more likely to worry about their asthma. Asthma affected the daily living of high SABA users. *Asthma control*: High SABA users had a higher likelihood to have uncontrolled asthma based on GINA-defined criteria than SABA non-users. On the other hand, SABA over-users were more likely to describe their asthma as somewhat controlled. *Asthma knowledge*: High SABA users perceived that they had enough information to manage their asthma. *Risk perceptions*: High SABA users had low risk of perceptions. *The use of SABA*: High SABA users felt embarrassed to use and carry their inhalers in public. Besides, more high SABA users are reported to believe SABA could prevent asthma flare-ups and attacks. On the other hand, reliever over-users may own multiple inhalers.	Questionnaires response relied on patient reporting, patients’ diagnosis could not be confirmed, response rate is unknown, etc.	Exploring the socially desirable response which tend to be provided by asthma patients. Understanding the medication use and a variety of measures in primary care.

### Perceptions on health status

Two studies by Hong et al. (2006) and Azzi et al. (2022) have reported data about perceptions of health status [50, 52]. A five-point scale (‘excellent, very good, good, fair, and poor) was used in the study by Hong et al. (2006), while the ratings of ‘excellent/very good, good or fair/poor’ were used in the study by Azzi et al. (2022) [50, 52].

According to Hong et al. (2006), fewer SABA inappropriate users (8.0%) rated their overall health as ‘excellent’ compared to appropriate users (13.4%) [52]. This is consistent with the findings reported by Azzi et al. (2022) that had a smaller number of high SABA users (44.6%), who rated their overall health as ‘excellent/very good’ compared with non-high SABA users (63.4%) [50]. In reference to the above findings, high SABA users were less likely to rate their health status as ‘excellent’.

### Perceptions and attitudes towards the impact of asthma

Three studies from the United Kingdom, the United States, and Australia have reported data on perceptions and attitudes towards the impact of asthma [50, 52, 53]. The patients’ perceptions and attitudes were reported qualitatively in the study by Cole et al. (2013) [53]. Hong et al. (2006) have identified that patients self-reported five types of functional limitations, which included instrumental activities of daily living (using telephones or taking medicines), activities of daily living (dressing or bathing) walking, social functioning (work or attending classes) and cognitive functions (confusion or memory loss) [52]. On the other hand, the impact of asthma was assessed by the ‘yes’ or ‘no’ answer in the questionnaire study by Azzi et al. (2022) [50].

Both the high and low SABA users had reduced employment opportunities, limited social activities, impaired mental health due to the frightening asthma attacks as well as suffered from stigmatization [53]. Similar outcomes were reported whereby 46.6% of SABA over-users felt that asthma had restricted their daily activities and 25.6% of SABA over-users claimed that asthma affected their sex life [50]. Similarly, according to Hong et al. (2006), more SABA-inappropriate users claimed that asthma caused functional limitations than SABA-appropriate users [52]. This is especially true when SABA-inappropriate users (29.8%) claimed that asthma limited their walking compared to SABA-appropriate users (15.8%) [52]. All three studies acknowledged that asthma had affected the patients’ daily activities both socially and physically [50, 52, 53].

### Perceptions towards asthma control

Two studies from Australia and a study from the United Kingdom have published data on perceptions towards asthma control [50, 51, 53]. Respondents were interviewed and rated their perceptions towards asthma control as ‘mild, moderate, or moderate-to-severe in the study by Cole et al. (2013) [53]. The GINA-defined criteria (‘well-controlled, partly controlled, uncontrolled’) were used to assess the level of asthma control in the study by Azzi et al., (2019) [51]. Besides, Azzi et al. (2022) reported on the level of asthma control from both the patients’ perceptions (‘well controlled’, ‘somewhat controlled’ or ‘poor control’) and the assessment by using GINA-defined criteria (‘well-controlled, ‘partly controlled’, ‘uncontrolled’) [50].

From the perspective of patients’ perceptions of their level of asthma control, SABA over-users (43.7%) perceived that their asthma was somewhat controlled, whereas the majority of the non-SABA over-users (80.6%) perceived that their asthma was well-controlled [50]. Similar perceptions were reported in the study conducted by Cole et al. (2013), which stated that high SABA users claimed that they have poor control of asthma symptoms [53].

As per the level of asthma control assessed using GINA-defined criteria, SABA over-users were more likely (59.2%) to have uncontrolled asthma [50]. Similar findings were reported in another study by Azzi et al. (2019) which stated that a significantly higher number of SABA over-users (59.0%) had uncontrolled asthma than non-SABA over-users (15.4%) [51].

### Perceptions towards asthma knowledge

Studies by Blakeston et al. (2021) and Azzi et al. (2022) have reported on the perceptions towards asthma knowledge [50, 54]. Respondents were interviewed regarding their perceptions towards asthma knowledge in the study by Blakeston et al. (2021) and were required to answer ‘yes’ or ‘no’ to the questionnaire in the study by Azzi et al. (2022) [50, 54]. The SABA over-users claimed that they had sufficient information to cope with their asthma, but they also expressed the need to look for more information [50]. However, contrasting findings were reported by Blakeston et al. (2021) who stated that frequent reliever users generally did not know that SABA overuse may worsen their asthma control [54].

### Risk perceptions

Two studies from Australia have reported the findings on risk perception towards the use of SABA [50, 51]. Both studies required the respondents to answer ‘yes’ or ‘no’ to the questions on risk perceptions. According to Azzi et al. (2022), more SABA over-users (22.7%) considered SABA was safe to use than non-SABA over-users (8.2%), whereas more SABA over-users (49.5%) were worried about the side effects caused by long term therapy of preventer medication than non-SABA over-users (37.8%) [50]. Another study by Azzi et al. (2019) stated that more SABA over-users (43.3%) experienced side effects such as dry mouth, palpitations, tremors, chest tightness, muscle cramps, or headache than non-SABA over-users (30.9%) [51].

### Perceptions, attitudes, and behaviors towards the use of short-acting β_2_-agonists

Studies from Australia, Canada, France, Germany, Japan, the United Kingdom, and the United States have published data regarding the perceptions, attitudes, and behaviors towards the use of SABA [50, 51, 53, 54]. The studies by Cole et al. (2013) and Blakeston et al. (2021), conducted qualitative interviews with the respondents regarding their perceptions, attitudes, and behaviors towards the use of SABA [53, 54]. On the other hand, respondents were requested to answer ‘yes’ or ‘no’ in the questionnaire study by Azzi et al. (2019) and Azzi et al. (2022) [50, 51].

According to Blakeston et al. (2021), SABA users were psychologically attached to SABA relievers due to the immediate relief effects of the medicines [54]. This is in concordance with the results of Cole et al. (2013) which stated that SABA over-users most likely had multiple reliever inhalers in their surrounding area to ensure that they received the ‘quick fix’ immediately when symptoms attacked [53].

Azzi et al. (2022) stated that 54.9% of SABA over-users even took their relievers to prevent asthma attacks [50]. Similar results were reported by Azzi et al. (2019) whereby non-SABA over-users (82.1%) were more likely to use their reliever inhalers than SABA over-users (60.6%) [51]. In comparison, all the above findings suggested that asthma patients had an over-reliance on SABA medicines due to their quick relief effects. Meanwhile, SABA over-users may feel embarrassed, carrying (21.3%) and using (29.2%) their reliever inhalers in the public area [50]. Similar findings were reported by Cole et al. (2013) in the United Kingdom whereby SABA over-users felt embarrassed about their relievers’ usage [53].

## Discussion

This review offers a thorough search of studies between the years 2000 and 2023 regarding the perceptions, attitudes, and behaviors towards the use of SABA. The final list includes five studies, and these studies show perceptions, attitudes, and behaviors of SABA users among consumers and patients in Australia, Canada, France, Germany, Japan, the United Kingdom, and the United States [50–54].

It is evident that most SABA over-users were less likely to perceive that they have ‘excellent’ health status and well-controlled asthma [50, 51, 53]. This is so as SABA overuse was correlated with poor health outcomes such as the greater risk of asthma exacerbations, asthma-related mortality, and outpatient consultations [15]. Also, patients with well-controlled asthma should not need to administer their relievers more than twice a week, while those who used the relievers thrice and above per week could be considered SABA over-users [1]. This is in accordance with the study by Winterstein and Hartzema (2005) which stated that SABA overuse led to uncontrolled asthma [56]. It is a fact that SABA overuse increases the chance of airways hyper-responsiveness and masks the real disease progression [57].

Most SABA over-users perceived that asthma has impacted their quality of life [50, 52, 53]. Similar findings showed that most severe asthma patients have functional limitations, that have restricted them from physical activities, social life, and workplace productivity is impacted [58–61].

Owing to the need of SABA users to deal with their symptoms and treatments, most suffered from asthma restrictions and have a poor health-related quality of life [62, 63]. Meanwhile, the above systematic review has shown that most SABA users did not acknowledge that SABA overuse could worsen their asthma and they need more information to cope with their asthma symptoms [50, 54].

This might be due to the lack of disease education or health literacy which subsequently contributes to the issue of over-reliance on SABA relievers [64]. There was evidence that limited health literacy contributes to patients having insufficient asthma medication knowledge. This also leads to difficulty in mastering the techniques to administer metered-dose inhalers [65]. The similar findings were reported by Thai and George (2010), which stated that asthma patients with limited health literacy are associated with a greater frequency to visit the emergency department as their asthma worsens [66]. Limited health literacy caused asthma patients to fail to self-manage their asthma as they might misunderstand the prescription instruction [67]. Subsequently, this may lead to more restrictions in their life.

It is evident that most SABA over-users were concerned about the safety and side effects experienced by the use of SABA medicines [50, 51]. This is in concordance with a study by Foster et al. (2020) which indicated that asthma patients who had replaced their SABA inhalers with budesonide-formoterol inhalers perceived that the side effects of SABA inhalers were lesser than the latter. However, scientific evidence showed less association between the use of budesonide-formoterol inhalers with asthma exacerbations [68]. Meanwhile, more SABA over-users were also concerned about the safety of long-term preventive medicines as they worry about the development of dependence on long-term treatment [69–72].

From the above findings, it is noticed that most SABA over-users had psychological linkage towards SABA due to the immediate relieving effects of the medicine [50, 51, 53, 54]. Most asthma patients would like their asthma symptoms to be relieved immediately [73, 74]. The emotional attachment might be developed during the initial phase of treatment when asthma patients were to keep and use their SABA inhalers whenever their symptoms worsened without a preventer medication [3]. As a result, patients may perceive that treating the symptoms alone would be workable and learn that SABA medicines work well to relieve their symptoms immediately [75]. However, SABA monotherapy was no longer recommended even for patients with mild asthma as it can increase asthma-related deaths and asthma exacerbations [1].

### Strengths, limitations, and future recommendations

This is the first systematic review, which recorded the perceptions, attitudes, and behaviors towards the use of SABA which comprises both qualitative and quantitative research articles. This systematic review has several limitations, as it included studies that were published in the English language only. Besides, the number of settings, study samples, and study designs employed in all the included studies were relatively small and limited. Despite these limitations, the review reported important insights into perceptions, attitudes, and behaviors towards the use of SABA. But the quality assessment checklists showed that the articles included are of good quality.

It is recommended that future research could report the perceptions, attitudes, and behaviors of SABA users in the Asia region. Additionally, future research could explore the perceptions, attitudes, and behaviors of healthcare professionals such as general practitioners’, community pharmacists’ or nurses’ perspectives. Moreover, the findings of this systematic review could be employed to develop SABA prescription guidelines that are more tailored to the asthma patients’ mindset.

## Conclusions

The systematic review highlighted that SABA over-users were less likely to describe their health status and asthma control as ‘excellent’ as they perceived that asthma had restricted their daily lives and were concerned about the side effects of SABA. Most SABA over-users did not know that frequent SABA usage would worsen their asthma control, and they exhibited psychological linkage towards the use of SABA. Collaborative efforts between policymakers, healthcare professionals and patients are needed to improve SABA prescribing practices and usage. This is in order to optimize asthma medicines management.

## Supporting information

S1 Checklist PRISMA 2020 checklist (based on the document of ‘Manuscript’). (DOCX)

S1 Table Summary of search strategies in different databases. (PDF)

S2 Table The critical appraisal of qualitative studies by using the JBI checklist for qualitative research (Lockwood et al., 2015). (PDF)

S3 Table The critical appraisal of cross-sectional studies by using the JBI checklist for analytical cross-sectional studies (Moola et al., 2020). (PDF)

S1 Appendix The critical appraisal of qualitative studies by using the JBI checklist for qualitative research (Lockwood et al., 2015). (DOCX)

S2 Appendix The critical appraisal of cross-sectional studies by using the JBI checklist for analytical cross-sectional studies (Moola et al., 2020). (DOCX)
