# Supplementary material for: Perceptions, attitudes, and behaviors of asthma patients towards the use of short-acting β2-agonists: A systematic review
Source: PLoS One. 2023 Apr 20;18(4):e0283876. doi: 10.1371/journal.pone.0283876 (PMC10118161; doi:10.1371/journal.pone.0283876)
Supplement: S1 Table — (PDF) [file pone.0283876.s002.pdf]

Database	Search strategies
PubMed	("percept"[All Fields] OR "perceptibility"[All Fields] OR "perceptible"[All Fields] OR "perception"[MeSH Terms] OR "perception"[All Fields] OR "perceptions"[All Fields] OR "perceptional"[All Fields] OR "perceptive"[All Fields] OR "perceptiveness"[All Fields] OR "percepts"[All Fields] OR ("percept"[All Fields] OR "perceptibility"[All Fields] OR "perceptible"[All Fields] OR "perception"[MeSH Terms] OR "perception"[All Fields] OR "perceptions"[All Fields] OR "perceptional"[All Fields] OR "perceptive"[All Fields] OR "perceptiveness"[All Fields] OR "percepts"[All Fields]) OR ("belief s"[All Fields] OR "culture"[MeSH Terms] OR "culture"[All Fields] OR "belief"[All Fields] OR "beliefs"[All Fields]) OR ("belief s"[All Fields] OR "culture"[MeSH Terms] OR "culture"[All Fields] OR "belief"[All Fields] OR "beliefs"[All Fields]) OR ("comprehension"[MeSH Terms] OR "comprehension"[All Fields] OR "understand"[All Fields] OR "understanding"[All Fields] OR "understands"[All Fields] OR "understandability"[All Fields] OR "understandable"[All Fields] OR "understandably"[All Fields] OR "understandings"[All Fields]) OR ("comprehension"[MeSH Terms] OR "comprehension"[All Fields] OR "understand"[All Fields] OR "understanding"[All Fields] OR "understands"[All Fields] OR "understandability"[All Fields] OR "understandable"[All Fields] OR "understandably"[All Fields] OR "understandings"[All Fields]) OR ("attitude"[MeSH Terms] OR "attitude"[All Fields] OR "attitudes"[All Fields] OR "attitude s"[All Fields]) OR ("attitude"[MeSH Terms] OR "attitude"[All Fields] OR "attitudes"[All Fields] OR "attitude s"[All Fields]) OR ("respond"[All Fields] OR "respondant"[All Fields] OR "respondants"[All Fields] OR "responded"[All Fields] OR "respondent s"[All Fields] OR "responder"[All Fields] OR "responders"[All Fields] OR "responding"[All Fields] OR "respondings"[All Fields] OR "responds"[All Fields] OR "surveys and questionnaires"[MeSH Terms] OR ("surveys"[All Fields] AND "questionnaires"[All Fields]) OR "surveys and questionnaires"[All Fields] OR "respondent"[All Fields] OR "respondents"[All Fields]) OR ("respond"[All Fields] OR "respondant"[All Fields] OR "respondants"[All Fields] OR "responded"[All Fields] OR "respondent s"[All Fields] OR "responder"[All Fields] OR "responders"[All Fields] OR "responding"[All Fields] OR "respondings"[All Fields] OR "responds"[All Fields] OR "surveys and questionnaires"[MeSH Terms] OR ("surveys"[All Fields] AND "questionnaires"[All Fields]) OR "surveys and questionnaires"[All Fields] OR "respondent"[All Fields] OR "respondents"[All Fields]) OR ("behavior"[MeSH Terms] OR "behavior"[All Fields] OR "behavioral"[All Fields] OR "behavioural"[All Fields] OR "behavior s"[All Fields] OR "behaviorally"[All Fields] OR "behaviour"[All Fields] OR "behaviourally"[All Fields] OR "behaviours"[All Fields] OR "behaviors"[All Fields] OR "pattern"[All Fields] OR "pattern s"[All Fields] OR "patternability"[All Fields] OR "patternable"[All Fields] OR "patterned"[All Fields] OR "patterning"[All Fields] OR "patternings"[All Fields] OR "patterns"[All Fields]) OR ("behavior"[MeSH Terms] OR "behavior"[All Fields] OR "behavioral"[All Fields] OR "behavioural"[All Fields] OR "behavior s"[All Fields] OR "behaviorally"[All Fields] OR "behaviour"[All Fields] OR "behaviourally"[All Fields] OR "behaviours"[All Fields] OR "behaviors"[All Fields] OR "pattern"[All Fields] OR "pattern s"[All Fields] OR "patternability"[All Fields] OR "patternable"[All Fields] OR "patterned"[All Fields] OR "patterning"[All Fields] OR "patternings"[All Fields] OR "patterns"[All Fields]) OR ("practicability"[All Fields] OR "practicable"[All Fields] OR "practical"[All Fields] OR "practicalities"[All Fields] OR "practicality"[All Fields] OR "practically"[All Fields] OR "practicals"[All Fields] OR "practice"[All Fields] OR "practice s"[All Fields] OR "practiced"[All Fields] OR "practices"[All Fields] OR "practicing"[All Fields]) OR ("practicability"[All Fields] OR "practicable"[All Fields] OR "practical"[All Fields] OR "practicalities"[All Fields] OR "practicality"[All Fields] OR "practically"[All Fields] OR "practicals"[All Fields] OR "practice"[All Fields] OR "practice s"[All Fields] OR "practiced"[All Fields] OR "practices"[All Fields] OR "practicing"[All Fields]) OR ("short-acting"[All Fields] AND "beta-2"[All Fields] AND ("agonist"[All Fields] OR "agonist s"[All Fields] OR "agonistic"[All Fields] OR "agonistically"[All Fields] OR "agonistics"[All Fields] OR "agonists"[MeSH Subheading] OR "agonists"[All Fields])) OR ("short-acting"[All Fields] AND "beta-2"[All Fields] AND ("agonist"[All Fields] OR "agonist s"[All Fields] OR "agonistic"[All Fields] OR "agonistically"[All Fields] OR "agonistics"[All Fields] OR "agonists"[MeSH Subheading] OR "agonists"[All Fields])) OR ("3 2 4 azidobenzamidino ethyl 5 hydroxyindole"[Supplementary Concept] OR "3 2 4 azidobenzamidino ethyl 5 hydroxyindole"[All Fields] OR "saba"[All Fields]) OR ("terbutaline"[MeSH Terms] OR

	"terbutaline"[All Fields] OR "terbutalin"[All Fields]) OR ("levalbuterol"[MeSH Terms] OR "levalbuterol"[All Fields] OR "albuterol"[All Fields] OR "albuterol"[MeSH Terms] OR "salbutamol"[All Fields]) OR ("levalbuterol"[MeSH Terms] OR "levalbuterol"[All Fields] OR "albuterol"[All Fields] OR "albuterol"[MeSH Terms] OR "salbutamol"[All Fields]) OR ("reliever"[All Fields] OR "relievers"[All Fields])) AND ((ffrft[Filter]) AND (fft[Filter]) AND (2000:2023[pdat]))
	((((((("percept"[All Fields] OR "perceptibility"[All Fields] OR "perceptible"[All Fields] OR "perception"[MeSH Terms] OR "perception"[All Fields] OR "perceptions"[All Fields] OR "perceptional"[All Fields] OR "perceptive"[All Fields] OR "perceptiveness"[All Fields] OR "percepts"[All Fields] OR ("percept"[All Fields] OR "perceptibility"[All Fields] OR "perceptible"[All Fields] OR "perception"[MeSH Terms] OR "perception"[All Fields] OR "perceptions"[All Fields] OR "perceptional"[All Fields] OR "perceptive"[All Fields] OR "perceptiveness"[All Fields] OR "percepts"[All Fields]) OR ("belief s"[All Fields] OR "culture"[MeSH Terms] OR "culture"[All Fields] OR "belief"[All Fields] OR "beliefs"[All Fields]) OR ("belief s"[All Fields] OR "culture"[MeSH Terms] OR "culture"[All Fields] OR "belief"[All Fields] OR "beliefs"[All Fields]) OR ("comprehension"[MeSH Terms] OR "comprehension"[All Fields] OR "understand"[All Fields] OR "understanding"[All Fields] OR "understands"[All Fields] OR "understandability"[All Fields] OR "understandable"[All Fields] OR "understandably"[All Fields] OR "understandings"[All Fields]) OR ("comprehension"[MeSH Terms] OR "comprehension"[All Fields] OR "understand"[All Fields] OR "understanding"[All Fields] OR "understands"[All Fields] OR "understandability"[All Fields] OR "understandable"[All Fields] OR "understandably"[All Fields] OR "understandings"[All Fields])) AND ("attitude"[MeSH Terms] OR "attitude"[All Fields] OR "attitudes"[All Fields] OR "attitude s"[All Fields])) OR ("attitude"[MeSH Terms] OR "attitude"[All Fields] OR "attitudes"[All Fields] OR "attitude s"[All Fields]) OR ("respond"[All Fields] OR "respondant"[All Fields] OR "respondants"[All Fields] OR "responded"[All Fields] OR "respondent s"[All Fields] OR "responder"[All Fields] OR "responders"[All Fields] OR "responding"[All Fields] OR "respondings"[All Fields] OR "responds"[All Fields] OR "surveys and questionnaires"[MeSH Terms] OR ("surveys"[All Fields] AND "questionnaires"[All Fields]) OR "surveys and questionnaires"[All Fields] OR "respondent"[All Fields] OR "respondents"[All Fields]) OR ("respond"[All Fields] OR "respondant"[All Fields] OR "respondants"[All Fields] OR "responded"[All Fields] OR "respondent s"[All Fields] OR "responder"[All Fields] OR "responders"[All Fields] OR "responding"[All Fields] OR "respondings"[All Fields] OR "responds"[All Fields] OR "surveys and questionnaires"[MeSH Terms] OR ("surveys"[All Fields] AND "questionnaires"[All Fields]) OR "surveys and questionnaires"[All Fields] OR "respondent"[All Fields] OR "respondents"[All Fields])) AND ("behavior"[MeSH Terms] OR "behavior"[All Fields] OR "behavioral"[All Fields] OR "behavioural"[All Fields] OR "behavior s"[All Fields] OR "behaviorally"[All Fields] OR "behaviour"[All Fields] OR "behaviourally"[All Fields] OR "behaviours"[All Fields] OR "behaviors"[All Fields] OR "pattern"[All Fields] OR "pattern s"[All Fields] OR "patternability"[All Fields] OR "patternable"[All Fields] OR "patterned"[All Fields] OR "patterning"[All Fields] OR "patternings"[All Fields] OR "patterns"[All Fields])) OR ("behavior"[MeSH Terms] OR "behavior"[All Fields] OR "behavioral"[All Fields] OR "behavioural"[All Fields] OR "behavior s"[All Fields] OR "behaviorally"[All Fields] OR "behaviour"[All Fields] OR "behaviourally"[All Fields] OR "behaviours"[All Fields] OR "behaviors"[All Fields] OR "pattern"[All Fields] OR "pattern s"[All Fields] OR "patternability"[All Fields] OR "patternable"[All Fields] OR "patterned"[All Fields] OR "patterning"[All Fields] OR "patternings"[All Fields] OR "patterns"[All Fields]) OR ("practicability"[All Fields] OR "practicable"[All Fields] OR "practical"[All Fields] OR "practicalities"[All Fields] OR "practicality"[All Fields] OR "practically"[All Fields] OR "practicals"[All Fields] OR "practice"[All Fields] OR "practice s"[All Fields] OR "practiced"[All Fields] OR "practices"[All Fields] OR "practicing"[All Fields]) OR ("practicability"[All Fields] OR "practicable"[All Fields] OR "practical"[All Fields] OR "practicalities"[All Fields] OR "practicality"[All Fields] OR "practically"[All Fields] OR "practicals"[All Fields] OR "practice"[All Fields] OR "practice s"[All Fields] OR "practiced"[All Fields] OR "practices"[All Fields] OR "practicing"[All Fields])) AND ("short-acting"[All Fields] AND "beta-2"[All Fields] AND ("agonist"[All Fields] OR "agonist s"[All Fields] OR "agonistic"[All Fields] OR "agonistically"[All Fields] OR "agonistics"[All Fields] OR "agonists"[MeSH Subheading] OR "agonists"[All Fields]))) OR ("short-acting"[All Fields] AND "beta-2"[All Fields] AND ("agonist"[All Fields] OR "agonist s"[All Fields] OR "agonistic"[All Fields] OR

	"agonistically"[All Fields] OR "agonistics"[All Fields] OR "agonists"[MeSH Subheading] OR "agonists"[All Fields])) OR ("3 2 4 azidobenzamidino ethyl 5 hydroxyindole"[Supplementary Concept] OR "3 2 4 azidobenzamidino ethyl 5 hydroxyindole"[All Fields] OR "saba"[All Fields]) OR ("terbutaline"[MeSH Terms] OR "terbutaline"[All Fields] OR "terbutalin"[All Fields]) OR ("levalbuterol"[MeSH Terms] OR "levalbuterol"[All Fields] OR "albuterol"[All Fields] OR "albuterol"[MeSH Terms] OR "salbutamol"[All Fields]) OR ("levalbuterol"[MeSH Terms] OR "levalbuterol"[All Fields] OR "albuterol"[All Fields] OR "albuterol"[MeSH Terms] OR "salbutamol"[All Fields]) OR ("reliever"[All Fields] OR "relievers"[All Fields])) AND ((ffrft[Filter]) AND (fft[Filter]) AND (2000:2023[pdat]))
Scopus	(((perception OR perceptions OR belief OR beliefs OR knowledge OR knowledges OR understanding OR understandings) OR (attitude OR attitudes OR respond OR responds) OR (behavior OR behaviors OR practice OR practices) OR short-acting AND beta-2 AND agonist OR short-acting AND beta-2 AND agonists OR saba OR terbutaline OR salbutamol OR albuterol OR reliever)) AND (LIMIT-TO (PUBYEAR , 2023) OR LIMIT-TO (PUBYEAR , 2022) OR LIMIT-TO (PUBYEAR , 2021) OR LIMIT-TO (PUBYEAR , 2020) OR LIMIT-TO (PUBYEAR , 2019) OR LIMIT-TO (PUBYEAR , 2018) OR LIMIT-TO (PUBYEAR , 2017) OR LIMIT-TO (PUBYEAR , 2016) OR LIMIT-TO (PUBYEAR , 2015) OR LIMIT-TO (PUBYEAR , 2014) OR LIMIT-TO (PUBYEAR , 2013) OR LIMIT-TO (PUBYEAR , 2012) OR LIMIT-TO (PUBYEAR , 2011) OR LIMIT-TO (PUBYEAR , 2010) OR LIMIT-TO (PUBYEAR , 2009) OR LIMIT-TO (PUBYEAR , 2008) OR LIMIT-TO (PUBYEAR , 2007) OR LIMIT-TO (PUBYEAR , 2006) OR LIMIT-TO (PUBYEAR , 2005) OR LIMIT-TO (PUBYEAR , 2004) OR LIMIT-TO (PUBYEAR , 2003) OR LIMIT-TO (PUBYEAR , 2002) OR LIMIT-TO (PUBYEAR , 2001) OR LIMIT-TO (PUBYEAR , 2000))
	(((perception OR perceptions OR belief OR beliefs OR knowledge OR knowledges OR understanding OR understandings) AND (attitude OR attitudes OR respond OR responds) AND (behavior OR behaviors OR practice OR practices) AND short-acting AND beta-2 AND agonist OR short-acting AND beta-2 AND agonists OR saba OR terbutaline OR salbutamol OR albuterol OR reliever))
PsychINFO	(perception OR perceptions OR belief OR beliefs OR understanding OR understandings) OR (attitude OR attitudes OR respond OR responds) OR (behavior OR behaviors OR practice OR practices) OR (short-acting beta-2 agonist OR short-acting beta-2 agonists OR SABA OR terbutaline OR salbutamol OR albuterol OR reliever)
	(perception OR perceptions OR belief OR beliefs OR understanding OR understandings) AND (attitude OR attitudes OR respond OR responds) AND (behavior OR behaviors OR practice OR practices) AND (short-acting beta-2 agonist OR short-acting beta-2 agonists OR SABA OR terbutaline OR salbutamol OR albuterol OR reliever)
CINAHL	(perception OR perceptions OR belief OR beliefs OR understanding OR understandings) OR (attitude OR attitudes OR respond OR responds) OR (behavior OR behaviors OR practice OR practices) OR (short-acting beta-2 agonist OR short-acting beta-2 agonists OR SABA OR terbutaline OR salbutamol OR albuterol OR reliever)
	(perception OR perceptions OR belief OR beliefs OR understanding OR understandings) AND (attitude OR attitudes OR respond OR responds) AND (behavior OR behaviors OR practice OR practices) AND (short-acting beta-2 agonist OR short-acting beta-2 agonists OR SABA OR terbutaline OR salbutamol OR albuterol OR reliever)

Cochrane database	(perception OR perceptions OR belief OR beliefs OR understanding OR understandings) OR (attitude OR attitudes OR respond OR responds) OR (behavior OR behaviors OR practice OR practices) OR (short-acting beta-2 agonist OR short-acting beta-2 agonists OR SABA OR terbutaline OR salbutamol OR albuterol OR reliever)
	(perception OR perceptions OR belief OR beliefs OR understanding OR understandings) AND (attitude OR attitudes OR respond OR responds) AND (behavior OR behaviors OR practice OR practices) AND (short-acting beta-2 agonist OR short-acting beta-2 agonists OR SABA OR terbutaline OR salbutamol OR albuterol OR reliever)
