# Supplementary material for: Perceptions, attitudes, and behaviors of asthma patients towards the use of short-acting β2-agonists: A systematic review
Source: PLoS One. 2023 Apr 20;18(4):e0283876. doi: 10.1371/journal.pone.0283876 (PMC10118161; doi:10.1371/journal.pone.0283876)
Supplement: S2 Table — (PDF) [file pone.0283876.s003.pdf]

Appraisal questions	(Cole et al., 2013)	(Blakeston et al., 2021)
1. Is there congruity between the stated philosophical perspective and the research method?	1	1
2. Is there congruity between the research method and the research question or objectives?	1	0
3. Is there congruity between the research method and the methods used to collect data?	1	1
4. Is there congruity between the research method and the representation and analysis of data?	1	1
5. Is there congruity between the research method and the interpretation of results?	1	1
6. Is there a statement finding the researcher culturally or theoretically?	0	0
7. Is the influence of the researcher on the research, and vice- versa, addressed?	1	0
8. Are participants, and their voices, represented?	1	1
9. Is the research ethical according to current criteria or, for recent studies, and is there evidence of ethical approval by an appropriate body?	1	1
10. Do the conclusions drawn in the research report flow from the analysis, or interpretation, of the data?	0	1
Quality percentage	$\frac{8}{10} \times 100\% = 80\%$	$\frac{7}{10} \times 100\% = 70\%$
Comment	High quality	High quality
