# Supplementary material for: Perceptions, attitudes, and behaviors of asthma patients towards the use of short-acting β2-agonists: A systematic review
Source: PLoS One. 2023 Apr 20;18(4):e0283876. doi: 10.1371/journal.pone.0283876 (PMC10118161; doi:10.1371/journal.pone.0283876)
Supplement: S3 Table — (PDF) [file pone.0283876.s004.pdf]

Appraisal questions	(Hong et al., 2006)	(Azzi et al., 2019)	(Azzi et al., 2022)
1. Were the criteria for inclusion in the sample clearly defined?	1	1	1
2. Were the study subjects and the setting described in detail?	1	1	1
3. Was the exposure measured in a valid and reliable way?	1	1	1
4. Were objective, standard criteria used for measurement of the condition?	1	1	1
5. Were confounding factors identified?	1	1	1
6. Were strategies to deal with confounding factors stated?	0	0	0
7. Were the outcomes measured in a valid and reliable way?<br><br>(This question could be ignored because the patients' self-reporting is acceptable in this literature review)	-	-	-
8. Was appropriate statistical analysis used?	1	1	1
Quality percentage	$\frac{6}{7} \times 100\% \approx 86\%$	$\frac{6}{7} \times 100\% \approx 86\%$	$\frac{6}{7} \times 100\% \approx 86\%$
Comment	High quality	High quality	High quality
