# Supplementary material for: Perceptions, attitudes, and behaviors of asthma patients towards the use of short-acting β2-agonists: A systematic review
Source: PLoS One. 2023 Apr 20;18(4):e0283876. doi: 10.1371/journal.pone.0283876 (PMC10118161; doi:10.1371/journal.pone.0283876)
Supplement: S1 Appendix — (DOCX) [file pone.0283876.s005.docx]

**Table 4**: The critical appraisal of qualitative studies by using the JBI checklist for qualitative research (Lockwood et al., 2015).

| Appraisal questions | (Cole et al., 2013) | (Blakeston et al., 2021) |
| --- | --- | --- |
| 1. Is there congruity between the stated philosophical perspective and the research method? | 1 | 1 |
| 1. Is there congruity between the research method and the research question or objectives? | 1 | 0 |
| 1. Is there congruity between the research method and the methods used to collect data? | 1 | 1 |
| 1. Is there congruity between the research method and the representation and analysis of data? | 1 | 1 |
| 1. Is there congruity between the research method and the interpretation of results? | 1 | 1 |
| 1. Is there a statement finding the researcher culturally or theoretically? | 0 | 0 |
| 1. Is the influence of the researcher on the research, and vice- versa, addressed? | 1 | 0 |
| 1. Are participants, and their voices, represented? | 1 | 1 |
| 1. Is the research ethical according to current criteria or, for recent studies, and is there evidence of ethical approval by an appropriate body? | 1 | 1 |
| 1. Do the conclusions drawn in the research report flow from the analysis, or interpretation, of the data? | 0 | 1 |
| Quality percentage | $\frac{8}{10}\times100\% =80\%$ | $\frac{7}{10}\times100\% =70\%$ |
| Comment | High quality | High quality |
