# Supplementary material for: Perceptions, attitudes, and behaviors of asthma patients towards the use of short-acting β2-agonists: A systematic review
Source: PLoS One. 2023 Apr 20;18(4):e0283876. doi: 10.1371/journal.pone.0283876 (PMC10118161; doi:10.1371/journal.pone.0283876)
Supplement: S2 Appendix — (DOCX) [file pone.0283876.s006.docx]

**Table 5**: The critical appraisal of cross-sectional studies by using the JBI checklist for analytical cross-sectional studies (Moola et al., 2020).

| Appraisal questions | Hee Hong et al. (2005) | Azzi et al. (2019) | Azzi et al. (2022) |
| --- | --- | --- | --- |
| 1. Were the criteria for inclusion in the sample clearly defined? | 1 | 1 | 1 |
| 1. Were the study subjects and the setting described in detail? | 1 | 1 | 1 |
| 1. Was the exposure measured in a valid and reliable way? | 1 | 1 | 1 |
| 1. Were objective, standard criteria used for measurement of the condition? | 1 | 1 | 1 |
| 1. Were confounding factors identified? | 1 | 1 | 1 |
| 1. Were strategies to deal with confounding factors stated? | 0 | 0 | 0 |
| 1. Were the outcomes measured in a valid and reliable way?   (This question could be ignored because the patients’ self-reporting is acceptable in this literature review) | - | - | - |
| 1. Was appropriate statistical analysis used? | 1 | 1 | 1 |
| Quality percentage | $\frac{6}{7}\times100\%\approx86\%$ | $\frac{6}{7}\times100\%\approx86\%$ | $\frac{6}{7}\times100\%\approx86\%$ |
| Comment | High quality | High quality | High quality |
